# CKMT1B is a potential prognostic biomarker and associated with immune infiltration in Lower-grade glioma

**DOI:** 10.1371/journal.pone.0245524

**Published:** 2021-01-19

**Authors:** Huadi Shi, Yuling Song, Zhi Song, Chun Huang

**Affiliations:** 1 Department of Internal Medicine, Zhanjiang Central Hospital, Guangdong Medical University, Zhanjiang, Guangdong Province, China; 2 Department of Oncology, Guangdong Medical University, Zhanjiang, Guangdong Province, China; Istituto Superiore di Sanità, ITALY

## Abstract

**Background:**

Lower-grade glioma (LGG) is the most common histology identified in glioma. CKMT1B has not been investigated in glioma. The purpose of this research was to investigate the prognostic value of CKMT1B and its correlation with immune infiltration in LGG.

**Methods:**

We used Gene Expression Profiling Interactive Analysis (GEPIA) to analyze the expression of CKMT1B in LGG. Univariate and multivariate Cox regression analyses were used to assess the effect of CKMT1B expression and screened variables on survival. The correlation between CKMT1B and immune infiltration was evaluated by TIMER and CIBERSORT. Moreover, the possible biological functions of CKMT1B were studied by GSEA. The statistical analysis was conducted by R software.

**Results:**

The expression of CKMT1B was significantly lower than the normal samples in LGG. Low expression of CKMT1B predicts a worse prognosis. Multivariate Cox analyses revealed that CKMT1B might be an independent favorable prognostic indicator. TIMER analysis revealed that CKMT1B expression level was related to immune infiltration. CIBERSORT analysis showed that CKMT1B expression was positively related to the infiltration level of activated mast cells and negatively related to macrophage M2 in LGG. Moreover, GESA showed that multiple cancer-related and immune-related gene sets were enriched in the low-CKMT1B group in the top 5 of the most significant differences.

**Conclusion:**

CKMT1B is a prognostic biomarker with potential applications and associated with immune infiltration in Lower-grade glioma.

## 1. Introduction

Glioma is a common primary intracranial neuroepithelial tumor according to the WHO classification of tumors of the central nervous system in 2007, grade I and grade II belong to low-grade gliomas (LGG), and most adults are grade II gliomas, mainly including astrocytoma oligoid glioma and oligoid astrocytoma [1]. At present, there are still many controversies about the treatment of LGG, and a relatively consistent view is that minimizing the tumor burden can effectively improve the survival rate of patients [2,3]. However, there was a tendency of recurrence and transformation to high-grade glioma, with high disability rate and fatality rate. It is crucial to find effective biomarkers and new therapeutic targets, which can help individualized treatment and prognostic prediction.

CKMT1B is a protein coding gene and co-expressed with other cytosolic creatine kinase in many cells, particularly in tissues with high-energy demands, including kidney, brain, testis, placenta, sperm [4]. CKMT1B has been reported as a major target of oxidative induced molecular damage in ischemic, cardiomyopathy and neurodegenerative diseases [5]. Notably, the overexpression of CKMT1B predicted a poor prognosis in breast cancer [6] and hepatocellular carcinoma [7]. The reason might be that high energy conversion leads to the failure to eliminate tumor cells by inducing apoptosis. However, it was reported that CKMT1B expression was greatly lower in high Gleason grade carcinoma compared with low grade carcinoma or normal prostate [8]. Another research also has shown that CKMT1B was frequently downregulated during oral carcinogenesis, leading to apoptosis arrest [9].

To our knowledge, CKMT1B has not been reported in LGG. In the present study, we evaluated the expression of CKMT1B in LGG by GEPIA. The gene expression and clinicopathology analysis were based on data obtained from TCGA to explore the role of CKMT1B in LGG. We used the online website CIBERSORT and TIMER to assess the correlation between CKMT1B and tumor-infiltrating immune cells. To further explore the biological pathway of CKMT1B involvement in the pathogenesis of LGG, Gene Set enrichment analysis (GSEA) was performed.

## 2. Materials and methods

### 2.1. Data mining of GEPIA and TCGA

The CKMT1B expression information (FPKM normalized) and clinical data were downloaded from the TCGA LGG cohort [10]. The collected clinicopathological data included gender, age, grade, survival status, and survival duration in days. Multivariate Cox regression analysis was used to assess the prognostic value of CKMT1B. In addition, the expression of CKMT1B in different tumor grades was compared using TCGA datasets. Our study was in line with the publication guidelines provided by TCGA. The GEPIA database (http://gepia.cancer-pk.cn/index.html) is an interactive web server developed by Beijing University for the integration and analysis of cancer expression spectrum data. It contains RNA sequencing expression data of 9,736 tumor samples and 8,587 normal samples from TCGA and GTEx. The function of GEPIA also includes several analysis modules, such as the analysis of survival analysis and correlation analysis of different tumor types or pathological stages [11]. The expression of CKMT1B between tumor and normal tissue was compared by GEPIA analysis. Next, we used the survival plots module of this online analysis tool to further analyze the relationship between the expression level of CKMT1B Gene and the Survival prognosis of LGG, and the selection criteria were shown in the Survival Plots: Gene:CKMT1B; Methods: Overall Survival; Group Cutoff: Median; Axis Units: moths; Datasets Selection: LGG.

### 2.2. Gene set enrichment analysis (GSEA)

The gene set enrichment analysis created a list of all genes related to the expression of CKMT1B. According to the median expression level of CKMT1B, CKMT1B was divided into high expression group and low expression group to explore the possible biological functions of CKMT1B by GO terms and KEGG pathways. The reference gene sets selected GOc5.all.v7.1.symbols.gmt and c2.cp.kegg.v7.1.symbols.gmt. The expression level of CKMT1B was used as phenotypic label, and multiple genome sequences were performed for each examination. The enrichment analysis was carried out according to the default parameter setting, and the number of random combinations was set as 1000. The nominal (NOM) P value and false discovery rate (FDR) q value ≤0.05 was taken as the significant enrichment gene sets.

### 2.3. Analysis of the relative abundance of tumor-infiltrating immune cells (TIICs)

CIBERSORT (http://cibersort.stanford.edu/) was a deconvolution algorithm based on gene expression. which has been widely used to analyze the correlation between gene expression in tumors and TIICs [12–14]. This analysis can be used to characterize the heterogeneity of cells according to the gene expression profile of complex tissues [15]. CIBERSORT used the inferred portion of the relative abundance of immune cell subsets to be accurate [16,17]. CIBERSORT can recognize immune cell types sensitively and accurately, so it can be further analyzed. The specific process is shown below. We downloaded the gene annotation matrix of 22 immune cell subtypes provided by the CIBERSORT network platform and calculated the P value of each sample based on the deconvolution algorithm. According to the median expression level of CKMT1B, CKMT1B was divided into high expression group and low expression group. The CIBERSORT could output the composition of infiltrating immune cells in each sample. Therefore, the relative proportion of various immune cells in high-CKMT1B expression group and low-CKMT1B expression group could be compared effectively. P < 0.05 were considered statistically significant.

### 2.4. Analysis of immune cell invasion, correlation of different genes and prognosis

Tumor immune estimation resource (TIMER) is a web server for comprehensive analysis of tumor-infiltrating immune cells [18] (https://cistrome.shinyapps.io/timer/). The abundance of six kinds of immune cells (B cells, CD4 + T cells, CD8 + T cells, neutrophils, macrophages and dendritic cells) can be estimated by statistical method and verified by pathological estimation. In addition, the “correlation” module can create scatterplots illustrating the expression of a pair of genes in a particular cancer type and also generates the Spearman correlation and estimated statistical significance, which can be adjusted by tumor purity (the proportion of cancer cells in the admixture) or age. We used this module to explore the correlation between CKMT1B expression and gene markers of immune infiltrating cells.

### 2.5. Validation of prognostic of CKMT1B and immune cell infiltration from CGGA database

The mRNA expression and clinical data were downloaded from the CGGA database (http://www.cgga.org.cn/) as validation set. The patients of validation set were divided into high-CKMT1B and low-CKMT1B expression group according to the cutoff value of CKMT1B expression from TCGA data set. The “survival" package of R software was used for Kaplan-Meier survival analysis. The evaluation of CKMT1B expression as an independent parameter was conducted by integrating the following clinical parameters into the univariate and multivariable Cox regression analysis: age, gender, grade, radiotherapy-status, chemotherapy-status, IDH-mutation status, MGMT-promoter methylated, 1p19q-codeletion, and CKMT1B expression. We also conducted the CIBERSORT to output the composition of infiltrating immune cells in each sample.

### 2.6. Statistical analysis

The listwise deletion technique was utilized to deal with any missing data, which excluded the entire sample from the investigation if any single value was absent. The Kaplan–Meier Plotter was used to evaluate the prognostic value of CKMT1B in LGG. The connection between the grade and CKMT1B was examined with the logistic regression. Clinical factors and the expression of CKMT1B were related to the overall survival using the multivariable Cox regression. The P value is converted to FDRs using the Benjamini Hochberg method. Data were examined with the R (version 3.6.1) and R Bioconductor packages. P < 0.05 was considered to have significant statistical significance.

## 3. Results

### 3.1. CKMT1B expression levels, survival results

**Fig 1:**
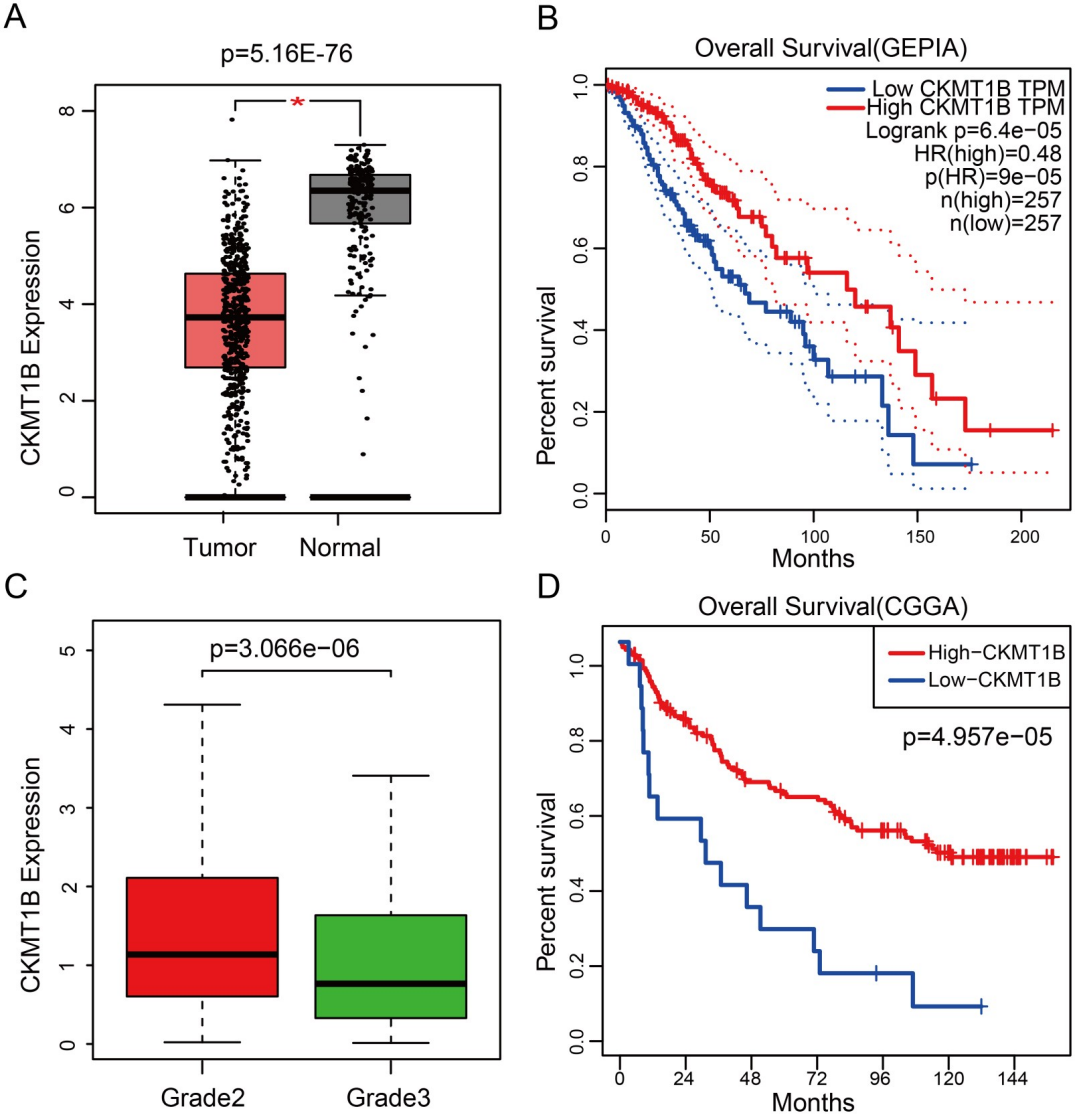
The role of CKMT1B level as prognostic factor for survival and the association with grade. (A) The expression of CKMT1B between normal and tumor tissues from GEPIA database. (B) Survival curve of differential CKMT1B expression were analyzed by GEPIA. (C) Differential expression of CKMT1B in different grade from TCGA database. (D) Survival curve of differential CKMT1B expression were analyzed in validation set from CGGA database. Abbreviations: CKMT1B, Creatine Kinase, Mitochondrial 1B; LGG, Lower-grade glioma; GEPIA, Gene Expression Profiling Interactive Analysis; CGGA, Chinese Glioma Genome Atlas.

CKMT1B expression levels in 518 tumor tissues were found to be a vast extent lower than that in 207 normal tissues (P = 5.16E-76, Fig 1A). We then explored connections between CKMT1B expression and overall survival. The Kaplan-Meier survival analysis demonstrated that LGG patients with higher levels of CKMT1B expression had a more good prognosis than those with a lower level of CKMT1B expression (P = 6.4e-05, Fig 1B).

### 3.2. Relationship between CKMT1B expression and clinicopathology

**Fig 2:**
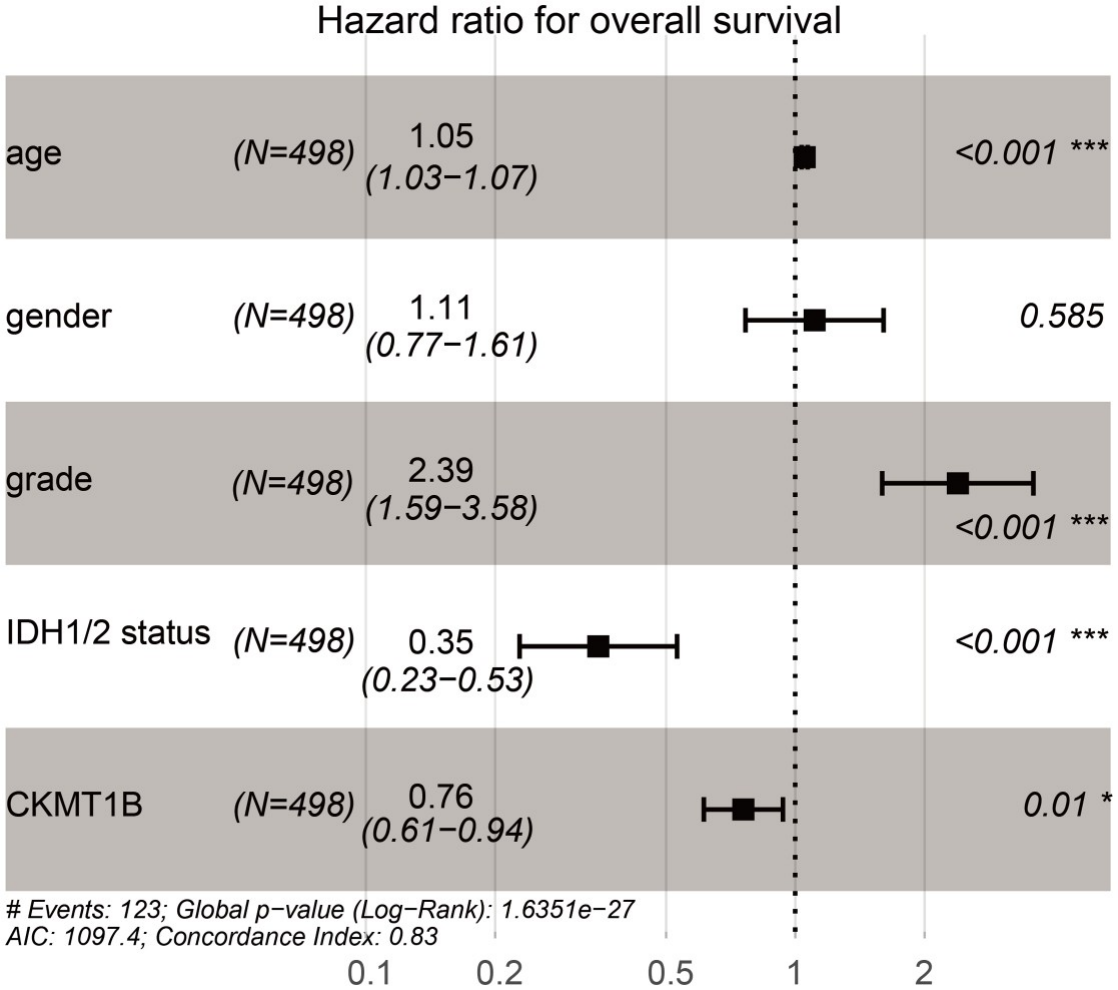
Multivariate Cox analysis of the correlation of CKMT1B expression with overall survival from TCGA database.

We assessed the relationship between CKMT1B expression levels and various clinicopathological parameters in LGG. TCGA involved 514 LGG samples, including CKMT1B expression data from patients with different clinical characteristics. LGG with grade II and III were included in our study cohort. There were 249 cases of grade II and 265 cases of grade III. The final survivors were followed for an average of 28.5 months, ranging from 0 to 214 months. Our principle for dealing with any missing data is that if a single value is missing, the entire sample is excluded from the survey. Using logistic regression analysis showed that the expression of CKMT1B was associated with tumor grade. LGG patients with low expression of CKMT1B were more likely to present a more advanced tumor grade (II vs III, odds ratio = 0.52, p-value<0.001, Fig 1C). As shown in Fig 2 and Table 1, multivariate Cox analysis uncovered that overall survival was significantly associated with age (HR = 1.05, P-value<0.001), grade (HR = 2.39, p-value<0.001), IDH1/2-mutation status (HR = 0.35, p-value<0.001), CKMT1B (HR = 0.76, p-value = 0.01) in TCGA datasets. These results suggest that CKMT1B may be an independent prognostic indicator for reducing cancer risk and prolonging patients’ overall survival time.

**Table 1:** Variables related to overall survival: Cox regression analysis.

Clinical Variables	Univariate analysis			Multivariate analysis		
	HR	95%CI	P-value	HR	95%CI	P-value
**age**	1.06	1.05–1.08	**0.000**	1.05	1.03–1.07	**0.000**
**gender**	1.07	0.75–1.53	0.707	1.11	0.77–1.61	0.585
**grade**	3.39	2.28–5.03	**0.000**	2.39	1.59–3.58	**0.000**
**IDH1/2 status**	0.19	.013–0.27	**0.000**	0.35	0.23–0.53	**0.000**
**CKMT1B**	0.70	0.57–0.86	**0.000**	0.76	0.61–0.94	**0.010**

Bold values indicate P < 0.05. HR. hazard ratio. CI. confidence interval.

### 3.3. GSEA analysis of CKMT1B

**Fig 3:**
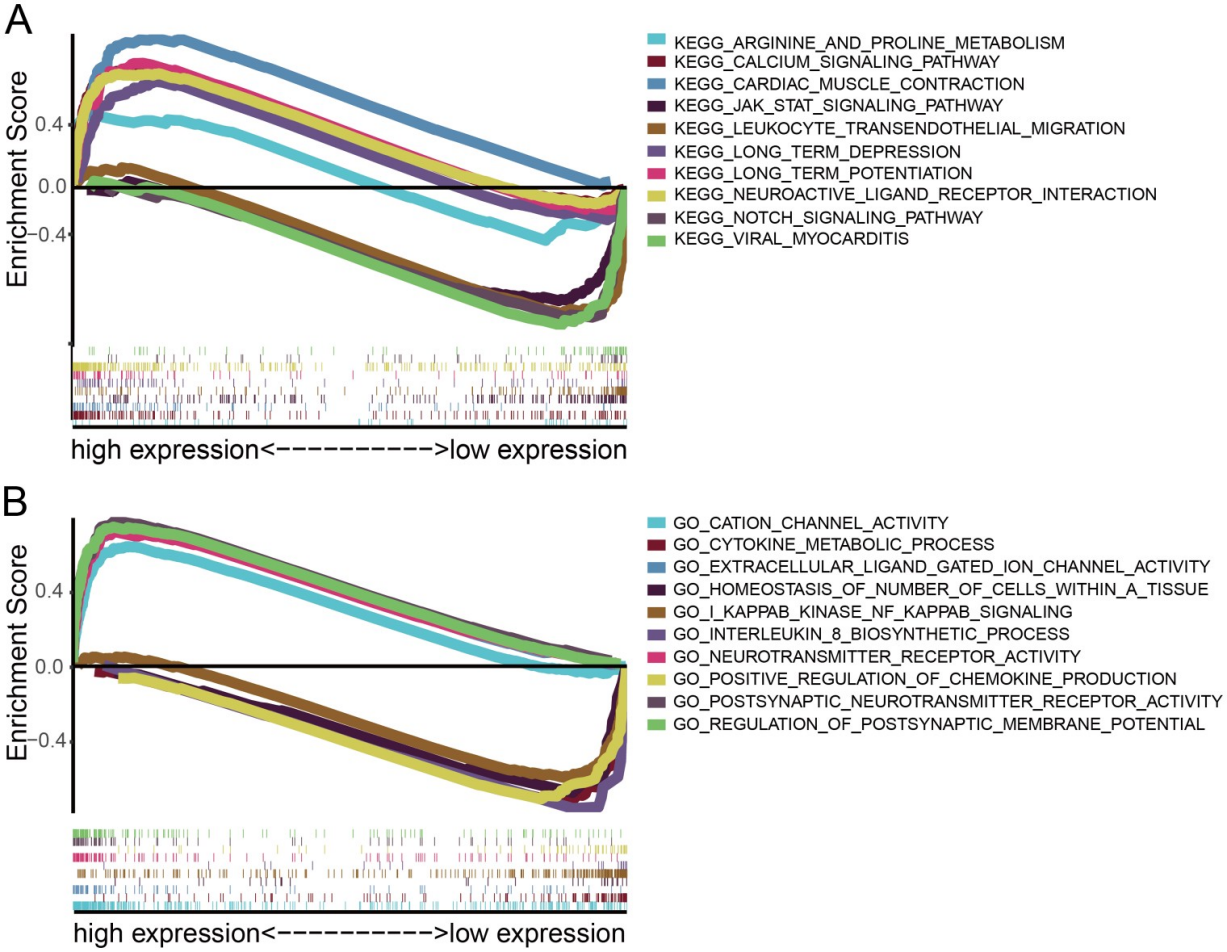
GSEA analysis of CKMT1B. (A) KEGG showed five signaling pathways in CKMT1B high and low expression groups by GSEA. (B) GSEA analysis revealed differential enrichment of genes in GO with CKMT1B high and low expression groups. (NES ≥2.0, NOM p-value < 0.05, FDR q-value <0.05). Abbreviations: GSEA, Gene Set Enrichment Analysis; GO, Gene Ontology; KEGG, Kyoto Encyclopedia of Genes and Genomes.

GO terms and KEGG pathway analyses were conducted to evaluate the potential biological functions of CKMT1B by GSEA. GSEA revealed significant differences in enrichment of GO terms and KEGG pathway in samples with high and low levels of CKMT1B. We only listed the top 5 GO terms and KEGG gene sets that were most significant in the high-CKMT1B and low-CKMT1B expression groups (NOM p-value < 0.01, FDR q-value < 0.05, Table 2). KEGG pathway analysis showed that multiple pathways were enriched in the high-CKMT1B group, including cardiac muscle contraction, neuroactive ligand receptor interaction, calcium signaling pathway, long term potentiation, long term depression and so on (NOM p-value < 0.05, FDR q-value < 0.05, Fig 3A). 5 KEGG items in the low expression group including leukocyte transendothelial migration, viral myocarditis, notch signaling pathway, antigen processing and presentation, JAK STAT signaling pathway (NOM p-value < 0.05, FDR q-value < 0.05, Fig 3A). GO terms analysis showed that postsynaptic neurotransmitter receptor activity, neurotransmitter receptor activity, regulation of postsynaptic membrane potential, insulin secretion involved in cellular response to glucose stimulus and extracellular ligand gated ion channel activity was enriched in the high-CKMT1B group (NOM p-value < 0.05, FDR q-value < 0.05, Fig 3B). Some immune-relative regulatory gene sets in GO terms were enriched in the low-CKMT1B group, including cytokine metabolic process, homeostasis of number of cells within a tissue, I kappab kinase NF kappab signaling, interleukin 8 biosynthetic process, positive regulation of chemokine production and so on (NOM p-value < 0.05, FDR q-value < 0.05, Fig 3B).

**Table 2:** Gene sets enriched in phenotype.

Gene set name	NES	P-value	FDR
**High-CKMT1B expression group**			
KEGG_CARDIAC_MUSCLE_CONTRACTION	2.14	0.000	0.003
KEGG_NEUROACTIVE_LIGAND_RECEPTOR_INTERACTION	1.94	0.000	0.029
KEGG_CALCIUM_SIGNALING_PATHWAY	1.89	0.002	0.038
KEGG_LONG_TERM_POTENTIATION	1.87	0.010	0.035
KEGG_LONG_TERM_DEPRESSION	1.86	0.002	0.032
GO_POSTSYNAPTIC_NEUROTRANSMITTER_RECEPTOR_ACTIVITY	2.44	0.000	0.000
GO_NEUROTRANSMITTER_RECEPTOR_ACTIVITY	2.39	0.000	0.000
GO_REGULATION_OF_POSTSYNAPTIC_MEMBRANE_POTENTIAL	2.37	0.000	0.000
GO_EXTRACELLULAR_LIGAND_GATED_ION_CHANNEL_ACTIVITY	2.36	0.000	0.000
GO_CATION_CHANNEL_ACTIVITY	2.36	0.000	0.000
**Low-CKMT1B expression group**			
KEGG_LEUKOCYTE_TRANSENDOTHELIAL_MIGRATION	-2.06	0.000	0.013
KEGG_VIRAL_MYOCARDITIS	-2.01	0.002	0.023
KEGG_NOTCH_SIGNALING_PATHWAY	-2	0.000	0.016
KEGG_ANTIGEN_PROCESSING_AND_PRESENTATION	-1.97	0.004	0.020
KEGG_JAK_STAT_SIGNALING_PATHWAY	-1.95	0.002	0.020
GO_CYTOKINE_METABOLIC_PROCESS	-2.25	0.000	0.003
GO_HOMEOSTASIS_OF_NUMBER_OF_CELLS_WITHIN_A_TISSUE	-2.24	0.000	0.002
GO_I_KAPPAB_KINASE_NF_KAPPAB_SIGNALING	-2.21	0.000	0.004
GO_INTERLEUKIN_8_BIOSYNTHETIC_PROCESS	-2.19	0.000	0.005
GO_POSITIVE_REGULATION_OF_CHEMOKINE_PRODUCTION	-2.19	0.000	0.004

FDR = false discovery rate, NES = normalized enrichment score. Gene sets with P-value<0.05 and FDR <0.05 are considered as significant.

### 3.4. Correlation between CKMT1B expression and TIICs composition

**Fig 4:**
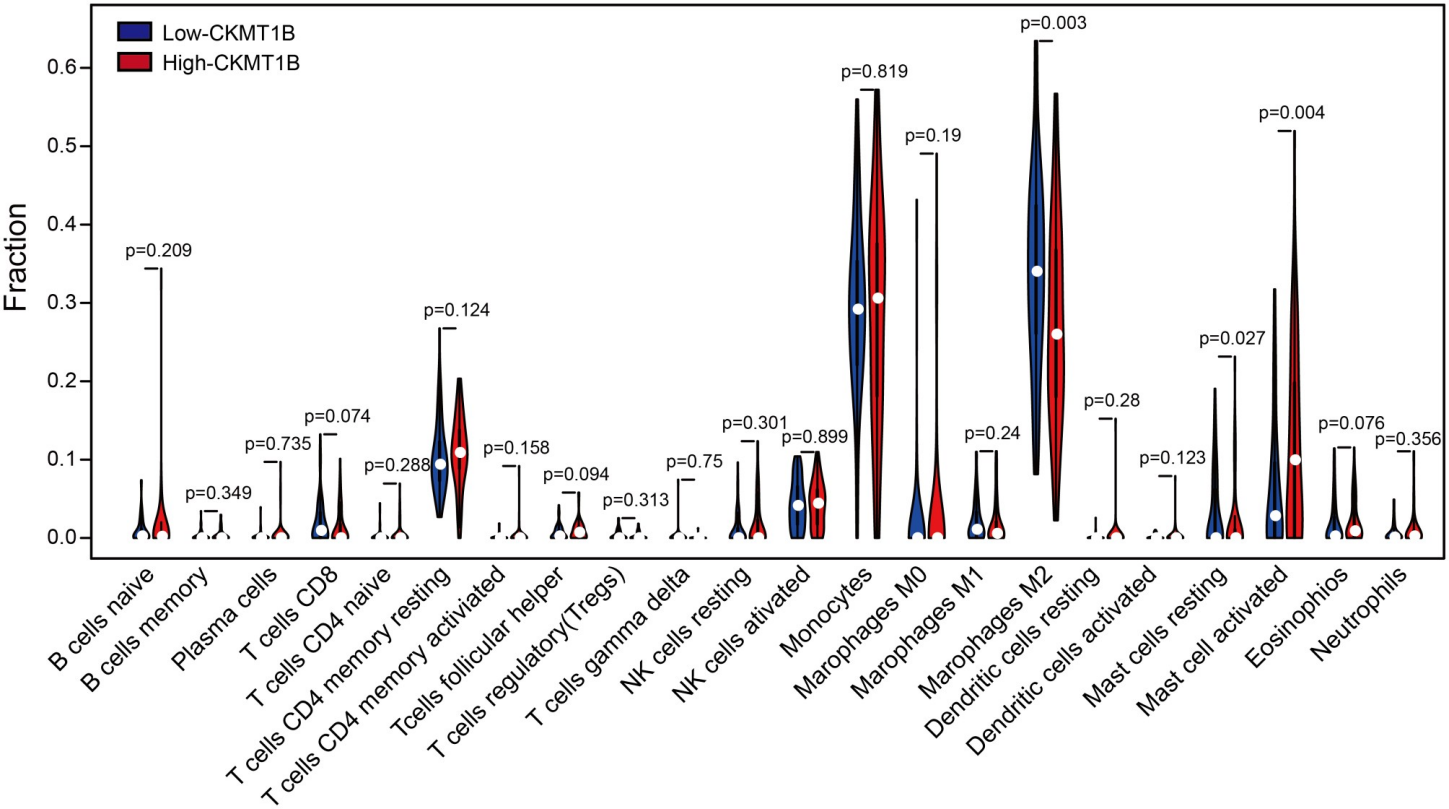
The varied proportions of 22 subtypes of immune cells in high and low CKMT1B groups in tumor samples. Horizontal and vertical axes respectively represent TIICs and relative percentages. Blue and red colors represent low and high CKMT1B expression groups, respectively.

We attempted to relate the CKMT1B expression to a variety of components of immune-infiltrating cells in the tumor microenvironment (TME) of LGG. A total of 514 sample data were downloaded from TCGA and were divided into high expression group and low expression group according to the median expression value of CKMT1B. The proportion of high expression group and low expression group meeting the screening criteria was 257 respectively. The difference in the proportion of 22 immune cells between the two groups was inferred by the CIBERSORT download gene expression profile. As shown in Fig 4, CKMT1B expression affects the infiltration level of M2 macrophages, activated mast cells and resting mast cells, among which M2 macrophages and activated mast cells are significantly different. M2 macrophages were decreased in high-CKMT1B expression group (p-value<0.01), while activated mast cells were increased (p-value<0.01).

### 3.5. Analysis of the TIMER

**Fig 5:**
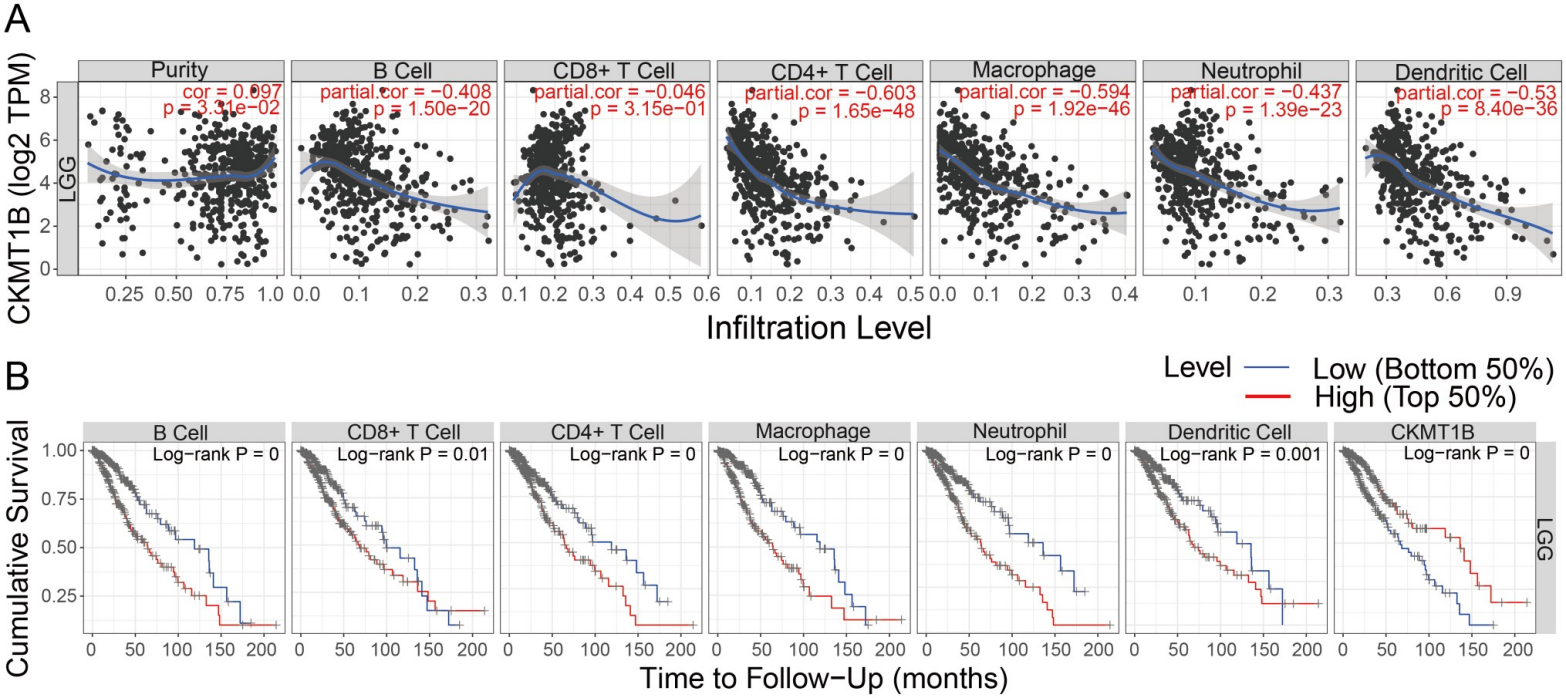
The TIMER analysis results. (A) CKMT1B expression level has significant negative correlations with infiltrating levels of B cell, CD8+ T cells, CD4+ T cells, dendritic cells, Macrophages and Neutrophils. (B) The B cells, CD4+ T cells, CD8+ T cells, dendritic cells, Macrophages and Neutrophils are correlated with the cumulative survival rate in LGG.

The TIMER algorithm was used to explore the possible correlation between CKMT1B expression and immune cell infiltration. Based on the LGG cohort of TCGA, we found that CKMT1B expression was positively correlated with tumor purity (r = 0.097, P<0.05). Conversely, CKMT1B expression had significantly negative correlations with infiltrating levels of B cells (r = -0.408, P<0.05), CD8+T cells (r = -0.046, P<0.05), CD4+T cells (r = -0.603, P<0.05), macrophages (r = -0.594, P<0.05), neutrophil (r = -0.437, P<0.05), and dendritic cells (r = -0.53, P<0.05) (Fig 5A). These findings indicate that CKMT1B is closely associated with immune cell infiltration in LGG. Additionally, the B cells, CD4+ T cells, CD8+ T cells, dendritic cells, Macrophages and Neutrophils are correlated with the cumulative survival rate in LGG (Fig 5B). We also included these immune cells for multivariate Cox analysis by TIMER. As shown in S1 Table, multivariate Cox regression analysis revealed that overall survival was significantly associated with age (HR = 1.064, P-value = 0.000), CKMT1B (HR = 0.773, p-value = 0.001).

### 3.6. Validation of prognostic of CKMT1B and immune cell infiltration

We used external dataset from the CGGA database for Kaplan-Meier survival analysis, Cox regression analysis and immune cell infiltration analysis. Consistent with our original data, high expression of CKMT1B predicts favorable prognosis in validation set (P = 4.957e−05, Fig 1D). As shown in S1 Fig, multivariate Cox analysis uncovered that overall survival was significantly associated with grade (HR = 3.76, p-value<0.001), 1p19q_codeletion_status (HR = 0.23, p-value<0.001), CKMT1B (HR = 0.75, p-value<0.001). It revealed that CKMT1B was an independent prognostic indicator in validation set. The CIBERSORT analysis of validation set from CCGA dataset showed that M2 macrophages were decreased in high-CKMT1B expression group (p-value = 0.223) and activated mast cells were increased (p-value = 0.173). The infiltration of M2 macrophages and mast cell were similar to the results from the TCGA dataset. However, the difference was not statistically significant (S2 Fig).

## 4. Discussion

In the present study, we investigated the potential role of CKMT1B in LGG and analyzed the expression of CKMT1B in a large cohort of human glioma patients for the first time. we explored the expression of CKMT1B as a prognostic biomarker for LGG. We observed a significantly lower expression of CKMT1B in the tumor compared with the normal samples by GEPIA. It is suggested that CKMT1B may play an important regulatory role in the progression of cancer. We also assessed the prognostic value of CKMT1B by GEPIA. We found that low expression of CKMT1B indicated a poor prognosis. To further investigate the potential mechanisms and relationships of CKMT1B expression in LGG, we downloaded the clinical and RNA-seq data from TCGA. Our results highlighted that the expression of CKMT1B was associated with tumor grade. LGG patients with low expression of CKMT1B were more likely to present a more advanced tumor grade. Moreover, CKMT1B can be a promising biomarker for cancer. Multivariate Cox regression analysis showed that CKMT1B expression was an independent factor affecting the prognosis of LGG patients. We also included these immune cells for multivariate Cox analysis by TIMER. It was further confirmed that CKMT1B predicted favorable prognosis and could act as an independent prognostic factor.

CKMT1B is a protein coding gene and co-expressed with other cytosolic creatine kinase in many cells, particularly in tissues with high-energy demands, including kidney, brain, testis, placenta, sperm [4]. CKMT1B has been reported as a major target of oxidative induced molecular damage in ischemic, cardiomyopathy and neurodegenerative diseases [5]. To our knowledge, CKMT1B has not been reported in LGG. The mechanism of CKMT1B in glioma cells is still unclear, but many studies have proved that CKMT1B was closely related to the occurrence and development of tumors. Qian et al. has proved that CKMT1B expression was correlated with a poor prognosis in breast cancer and might serve as a tumor marker. High CKMT1B expression promotes tumor growth by inhibiting apoptosis of tumor cells and down regulating mitochondrial apoptotic pathway proteins [6]. Uranbileg et al. found similar results in hepatocellular carcinoma. High expression of CKMT1B indicates highly malignant potential in hepatocellular carcinoma [7]. However, CKMT1B seems to play a different role in other cancers. It was reported that CKMT1B (uMtCK) expression was significantly decreased in prostate cancer tissues with higher Gleason scores, a measure of prostate cancer stage, compared with those with lower Gleason scores. CKMT1B expression was also significantly decreased in poorly differentiated prostate cancer tissues compared with well-differentiated cancer tissues [8]. The K-M survival curve analyses of 128 patients with prostate cancer showed that high-CKMT1B expression slightly reduced the PSA recurrence-free survival rate compared with patients with weak to moderate CKMT1B expression. Another research also has shown that CKMT1B was frequently downregulated during oral carcinogenesis, leading to apoptosis arrest [9]. Lan et al. found that CKMT1B could increase the sensitivity of CNE-1 to chemotherapy drug DDP by inhibiting STAT3 activation. The CNE-1 cell lines treated with overexpression CKMT1B had lower clonal formation rate, more obvious cell cycle arrest in G2/M phase and higher apoptosis rate [19]. These findings suggest that CKMT1B may have a dual role. On the one hand, CKMT1B acts as a tumor suppressor by promoting apoptosis of cancer cells. One possible mechanism is that CKMT1B reduces release of cytochrome C, eventually prevents activation of Caspase and the initiation of apoptosis [6]. On the other hand, it can also promote tumor progression by inhibiting apoptosis. It has been suggested that CKMT1B might induce apoptosis through mitochondrial permeability transition pore [9]. Our study also seems to support the role of CKMT1B as a tumor suppressor gene. We can’t explain the specific mechanism and cause of this paradoxical phenomenon for the moment. The effect of CKMT1B on apoptosis of cancer cells needs further study. Although the relationship between CKMT1B and LGG has not been explained in detail, based on our results and previous studies on CKMT1B, it is reasonable to believe that CKMT1B affects the development of the pathophysiological mechanisms of LGG.

To further explore the potential biological functions of CKMT1B in LGG, GO terms and KEGG pathway analyses were performed by GSEA. GSEA revealed significant differences in enrichment of GO terms and KEGG pathway in samples with high and low levels of CKMT1B. Specially, GESA analysis in the top 5 of the most significant differences has shown that multiple cancer-related and immune-related gene sets were enriched in the low-CKMT1B group, including notch signaling pathway, antigen processing and presentation, JAK STAT signaling pathway, cytokine metabolic process, interleukin 8 biosynthetic process, positive regulation of chemokine production and so on. As we all know, lots of evidence reveals that NOTCH signaling pathway [20,21] and JAK STAT signaling pathway [22,23] are all involved in the development of cancer. These findings suggest that CKMT1B may play an important role in the development of cancer. Low CKMT1B expression levels may influence mechanisms of tumorigenesis and tumor immunology in LGG progression. Our results show that low expression of CKMT1B is associated with a poor prognosis. Therefore, we believe that the lower expression of CKMT1B may play an important regulatory role in these oncogenic pathways for the worse prognosis of LGG.

Moreover, we found that many immune-related gene sets were significantly enriched in the low-CKMT1B expression group by GSEA. Therefore, in order to further analyze the correlation between CKMT1B and immune cell infiltration in LGG, we operated the online website TIMER which was a web server for comprehensive analysis of tumor-infiltrating immune cells [18]. Our results support the association of CKMT1B with prognosis and immune infiltration of LGG. In addition, CKMT1B expression had significantly negative correlations with infiltrating levels of B cells, CD8+T cells, CD4+T cells, macrophages, neutrophil and dendritic cells. These findings indicate that CKMT1B is closely associated with immune cell infiltration in LGG. Additionally, the B cells, CD4+ T cells, CD8+ T cells, dendritic cells, Macrophages and Neutrophils are correlated with the cumulative survival rate in LGG.

The interaction between immune cells and tumor cells in tumor microenvironment affects the occurrence, development and metastasis of cancer [24]. Tumor-associated macrophages (TAMs) is one of the most important immunosuppressive cell in tumor microenvironment (TME), mediating tumor progression by regulating TME [25,26]. In general, monocytes/macrophages can be polarized into M1-type or M2-type macrophages. M1 macrophages, also known as classically activated macrophages, are induced by pro-inflammatory (such as IFN-γ) and Immunostimulant cytokines (such as IL-12, IL-23). However, TAMs are considered to be more similar to M2 macrophages, which also known as substitution activated macrophages, activated by Th2 cytokines (such as IL4, IL-10, and IL-13) [27]. TAMs plays a crucial role in promoting tumor progression, including promoting tumor genesis and progression, forming immunosuppressive microenvironment to promote metastasis, establishing precancerous metastasis microenvironment, and promoting tumor angiogenesis [28–31]. Multiple cytokines produced by tumor cells can promote M2-type polarization of macrophages. Studies have found that CSF1 derived from colon cancer cells can promote the recruitment of macrophages and polarization of TAMs [32]. The polarization of TAMs mediated by tumor-derived microparticles promotes tumor progression [33]. In addition, mast cells in the tumor microenvironment not only participate in promoting tumor angiogenesis, but also play an important role in the immune response of anti-tumor [34–37]. In our study, an important conclusion was the correlation between CKMT1B expression and the level of immune infiltration in LGG. CIBERSORT analysis showed that CKMT1B expression was positively correlated with the infiltration level of activated mast cells and negatively correlated with the infiltration level of macrophage M2 in LGG. Similarly, the relationship between gene markers of different immune cells and CKMT1B expression suggests that CKMT1B plays an important role in regulating microenvironment of LGG. Our overall study results highlight the significant influence of CKMT1B on the immune infiltration of M2 macrophages and mast cells in LGG. Therefore, we believe that CKMT1B may have a potential influence on tumor immunity. The positive influence of CKMT1B in LGG may be related to the low density of M2 macrophages infiltration and the high density of mast cells.

This study still has some limitations. First, some clinical variables which may be confounders for CKMT1B were not included in the Cox regression analysis due to the large proportion of N/A (not available) values, such as radiotherapy-status, chemotherapy-status. Secondly, although the infiltration of M2 macrophages and mast cell from the validation set (CCGA dataset) were similar to the results from the TCGA dataset, the difference was not statistically significant. Thirdly, the molecular mechanism of CKMT1B affecting the prognosis of LGG patients and its significance for clinical translational therapy need to be further studied.

## 5. Conclusion

In conclusion, this is the first study to demonstrate the role of CKMT1B in LGG. CKMT1B is downregulated in LGG and related to the tumor grade. Meanwhile, Low expression of CKMT1B is associated with a poor prognosis and immune infiltration in glioma. In addition, the key pathway of LGG regulated by CKMT1B might be the notch signaling pathway, antigen processing and presentation, JAK STAT signaling pathway, cytokine metabolic process, interleukin 8 biosynthetic process and positive regulation of chemokine production. Finally, the results further suggest that an independent prognostic factor, high expression of CKMT1B mRNA, could be used to improve clinical outcomes in LGG patients. We strongly recommend further research on this topic to gradually refine the evidence for the biological effects of CKMT1B.

## Supplemental methods

The R scripts and packages used in the analysis are described in this section. The expression of CKMT1B in different tumor grades was compared using TCGA datasets and performed the function “boxpot()” from R language for visualization. The simplified code: boxplot (value ~ group, dataset). The function “wilcox.test()” was used to compare the two samples. The simplified code: wilcox.test (formula, data, subset); The “survival" package of R software was used for Kaplan-Meier survival analysis. The survival object is created using the “Surv()” function, as shown below: Surv(time, event). Then “survfit()” function was used to fit the survival function to the survival data object, and Kaplan-Meier survival curve was created. The simplified code: surfit(formula, data); The results of the GSEA and CIBERSORT analyses were visualized using”ggplot2”, ”vioplot” packages in R respectively. The “LIMMA” was selected as the differential expression analysis method in the GEPIA database. The “LIMMA” package of R software use false discovery rate (FDR) as the multiple statistical testing method to control the percentage of false positives in the resulting analysis. The calculation of FDR usually adopts Benjamini-Hochberg method. Multivariate Cox regression used “survival” and “survminer” packages of R3.6.1 for analysis and visualization. The function “coxph()” from the "survival" packages can be used to calculate the Cox proportional hazard regression model. The simplified format is as follows: coxph(formula, data, method). The survival object is created using the “Surv()” function. The function”ggforest()” from the “survminer” package creates forest plot for a Cox regression model fit. Hazard ratio estimates along with confidence intervals and p values are plotter for each variable.

## Supporting information

S1 Fig Multivariate Cox analysis of the correlation of CKMT1B expression with overall survival from CGGA database. (TIF)

S2 Fig The varied proportions of 22 subtypes of immune cells in high and low CKMT1B groups in tumor samples from CGGA database. Horizontal and vertical axes respectively represent TIICs and relative percentages. Blue and red colors represent low and high CKMT1B expression groups, respectively.
(TIF)

S1 Table Multivariate analyses of CKMT1B expression and other clinical factors as well as immune cells related to overall survival in LGG. (TIF)
