# Supplementary figures and images for: CKMT1B is a potential prognostic biomarker and associated with immune infiltration in Lower-grade glioma
Source: PLoS One. 2021 Jan 19;16(1):e0245524. doi: 10.1371/journal.pone.0245524 (PMC7815138; doi:10.1371/journal.pone.0245524)

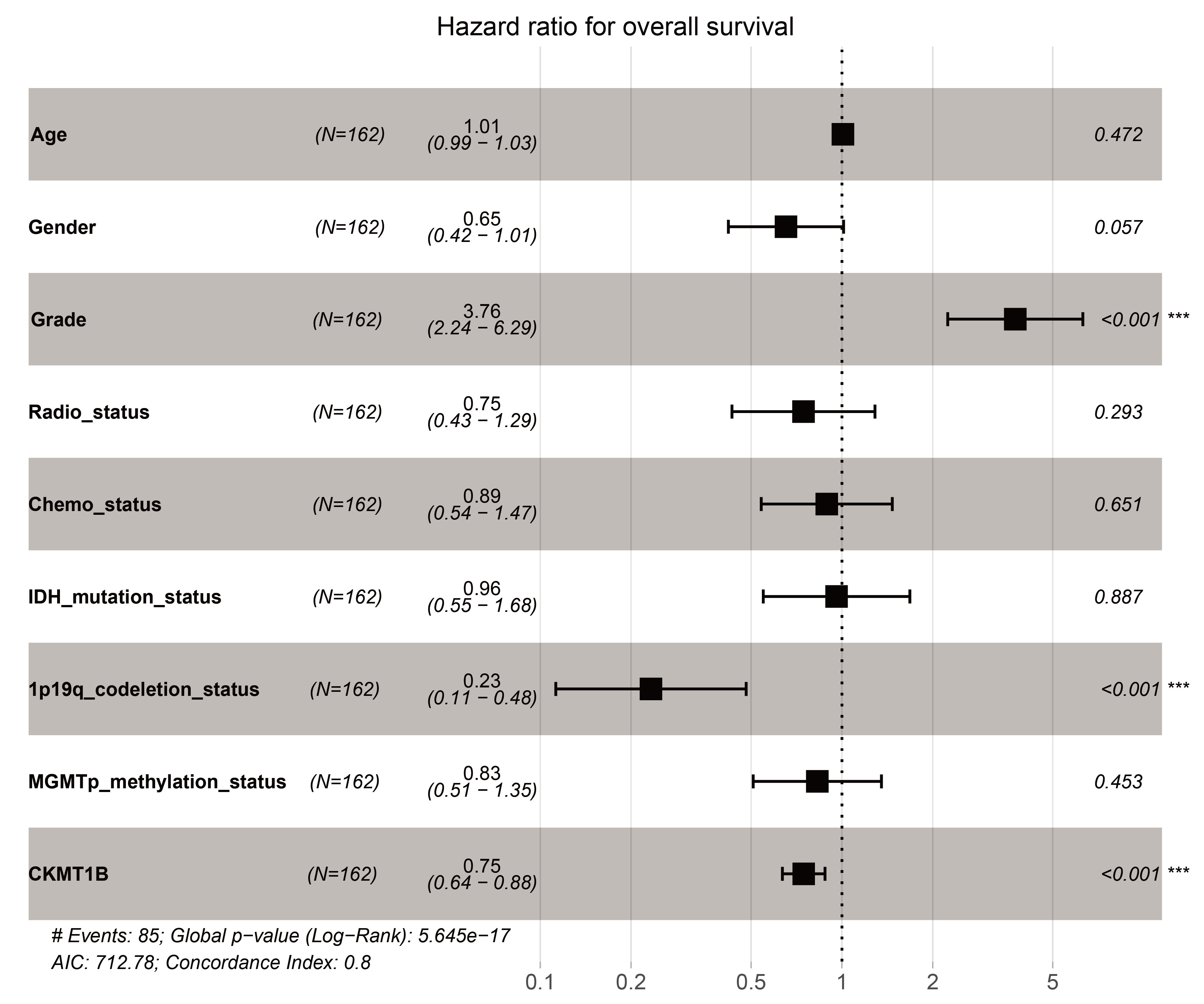

Supplement: S1 Fig — (TIF) [file pone.0245524.s001.tif]

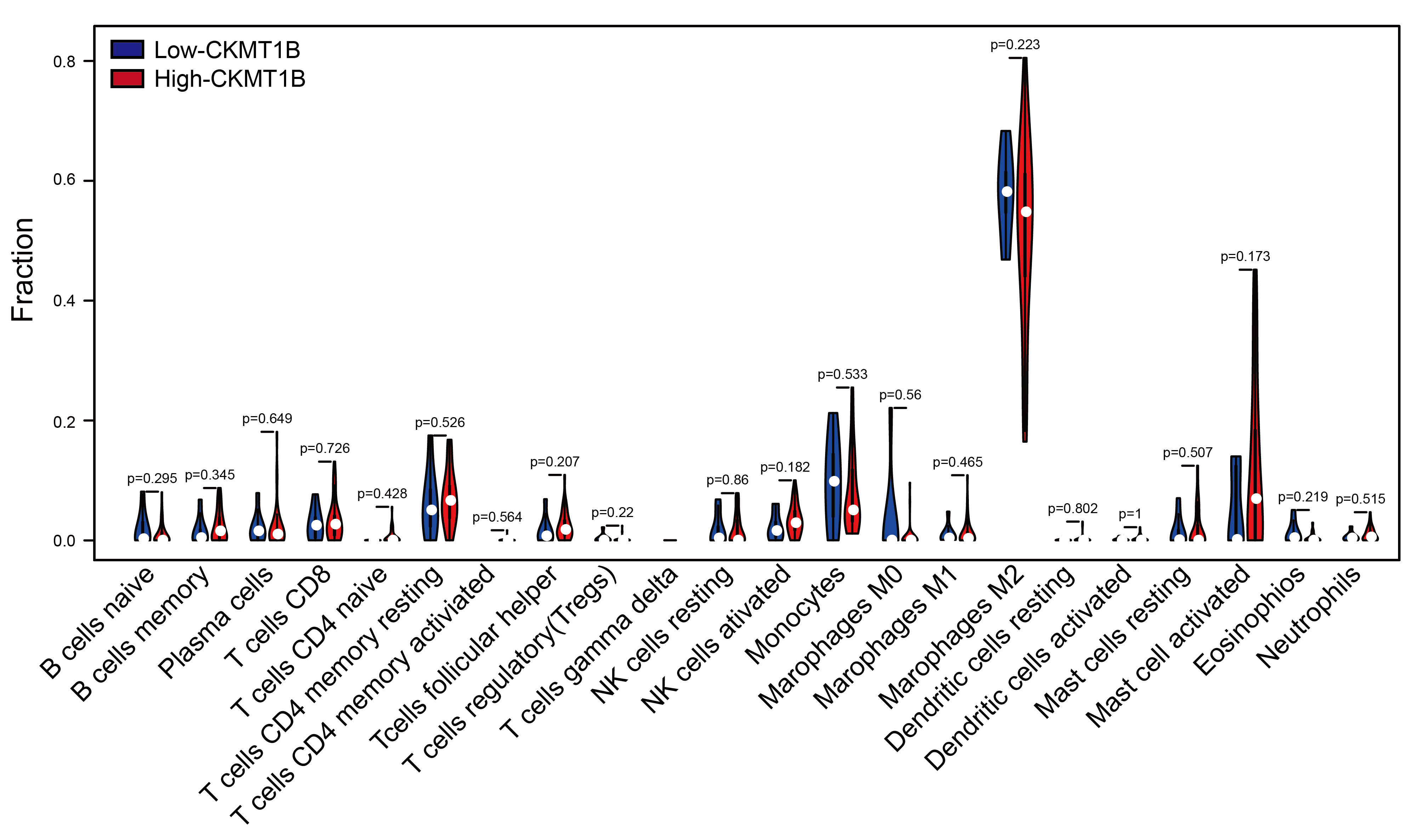

Supplement: S2 Fig — Horizontal and vertical axes respectively represent TIICs and relative percentages. Blue and red colors represent low and high CKMT1B expression groups, respectively. (TIF) [file pone.0245524.s002.tif]

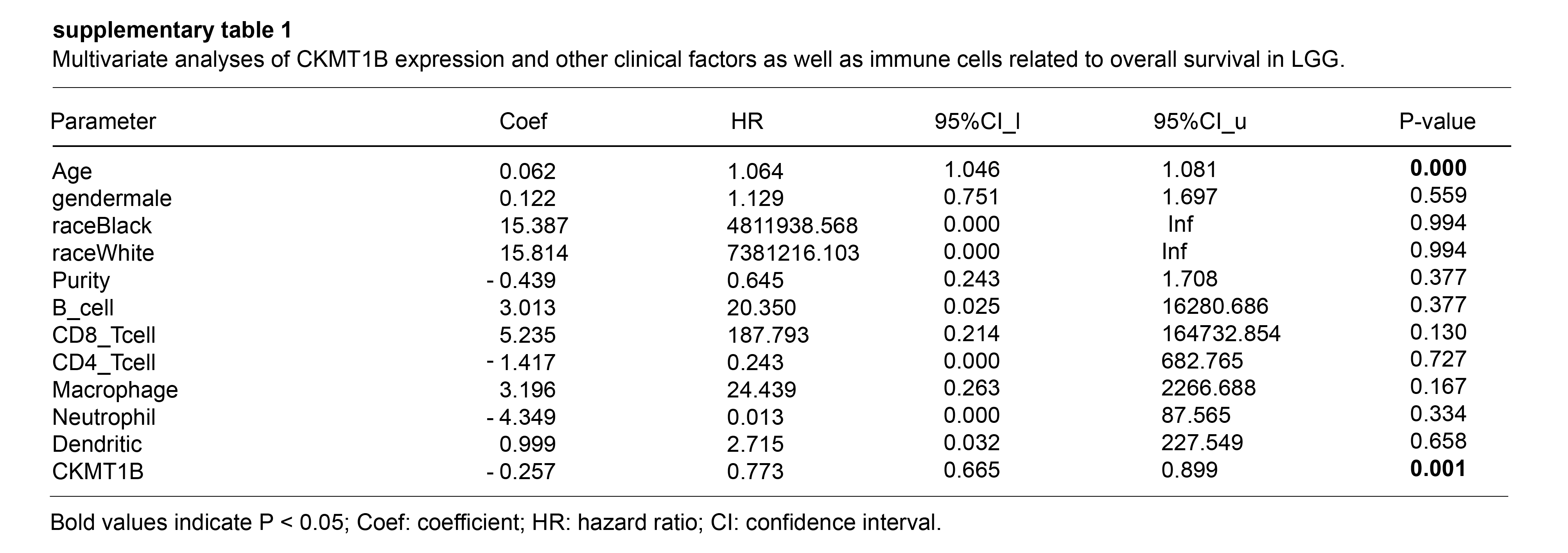

Supplement: S1 Table — (TIF) [file pone.0245524.s003.tif]
